# Comparative impact of bacitracin and select feed additives in the feeding program of Lohmann LSL-Lite pullets at the onset of lay through to 31 weeks of age

**DOI:** 10.1093/tas/txae013

**Published:** 2024-01-25

**Authors:** Elijah G Kiarie, Veronica Cheng, Zhigang Tan, Wenzhen Chen, Xiangyi Xu, Yu Peng, Haijun Liu, Zonghua Qin, Xianfeng Peng

**Affiliations:** Department of Animal Biosciences, University of Guelph, Guelph, ON N1G 2W1, Canada; Department of Animal Biosciences, University of Guelph, Guelph, ON N1G 2W1, Canada; Guangzhou Insighter Biotechnology Co., Ltd., Huangpu District, Guangzhou, Guangdong 510700, China; Guangzhou Insighter Biotechnology Co., Ltd., Huangpu District, Guangzhou, Guangdong 510700, China; Guangzhou Insighter Biotechnology Co., Ltd., Huangpu District, Guangzhou, Guangdong 510700, China; Guangzhou Insighter Biotechnology Co., Ltd., Huangpu District, Guangzhou, Guangdong 510700, China; Guangzhou Insighter Biotechnology Co., Ltd., Huangpu District, Guangzhou, Guangdong 510700, China; Guangzhou Insighter Biotechnology Co., Ltd., Huangpu District, Guangzhou, Guangdong 510700, China; Guangzhou Insighter Biotechnology Co., Ltd., Huangpu District, Guangzhou, Guangdong 510700, China

**Keywords:** *Aspergillus niger* probiotic, bacitracin, benzoic acid, egg production and quality at the onset of lay, gut health and metabolism, myristic acid

## Abstract

There are limited investigations on the role of feed additives in easing transition of pullets to egg production phase. We investigated the effects of supplementation of bacitracin methylene disalicylate (**BMD**) and select feed additives (myristic acid [**MA**], benzoic acid [**BA**], and *Aspergillus niger* probiotic [**PRO**]) in feeding program for pullets from the onset of lay through to 31 weeks of age (**woa**). Parameters measured included hen-day egg production (**HDEP**), feed intake (**FI**), feed conversion ratio (**FCR**), egg quality characteristics, ceca microbial activity, apparent retention of components, and plasma metabolites. A total of 1,200 Lohmann LSL Lite pullets were procured at 18 woa and placed in enriched cages (30 birds/cage) based on body weight (**BW**) and allocated to five diets. The diets were a basal diet formulated to meet specifications or basal mixed with either BMD, MA, BA, or PRO. Birds had free access to feed and water throughout the experiment. Between 18 and 20 woa, birds fed BMD ate a similar (*P* > 0.05) amount of feed to BA birds, but more (*P* = 0.0003) than birds fed basal, MA, or PRO diets. Basal birds had lower HDEP (*P* = 0.001) and lighter eggs (*P* < 0.0001) than birds fed any of the feed additives between 21 and 31 woa. The basal hens had a higher (*P* = 0.009) abundance of *Escherichia coli* than birds fed BMD, BA, and PRO diets. Consequently, BMD, BA, and PRO birds had a higher (*P* = 0.011) *Lactobacilli*: *E. coli* ratio (**LER**) than hens fed the basal diet. Specifically, relative to basal-fed hens, the LER of the BMD, MA, BA, and PRO hens was higher by 37%, 21%, 26%, and 45%, respectively. Moreover, birds fed PRO tended to have a higher concentration of ceca digesta acetic acid (*P* = 0.072) and a lower concentration of isobutyric acid (*P* = 0.096). In conclusion, supplementing pullet diets with broad-spectrum antibiotics or feed additives (MA, BA, and PRO) had a positive impact on FI, and egg production linked to modulation of indices of gut health. The results suggested supplementing feed additives in feeding programs for pullets at the onset of lay can bolster productivity outcomes.

## Teaser

Modulating gastrointestinal ecology through supplementation with feed additives at the onset of lay can bolster productivity outcomes linked to gut health and metabolism.

## INTRODUCTION

The transition from rearing to production is a very stressful period for the pullet with implication on lifetime egg production and livability (Leeson and Summers, 2005; Bain et al., 2016). This is because the pullets are subjected to many changes during this period including transportation, adaptation to a new environment (equipment and lighting regimen), and feed composition. Yet, peculiarities of the pullet at the point of lay are that she is yet to attain mature body weight (**BW**), exhibits low feed intake (**FI**), and has tremendous ability to attain peak production shortly after the first egg (Foster, 1968; Leeson and Summers, 2005; Anderson et al., 2013). As such at the beginning of lay, the pullet is not only adapting to her new environment, but she must support BW development amid looming peak egg production. Consequently, negative nutrient balances can occur and much so exacerbated if the point of lay body condition is suboptimal (Leeson and Summers, 2005; Widowski and Torrey, 2018; Hanlon et al., 2022).

In recognition of the critical role of pullet condition at onset lay and the subsequent impact on flock performance, significant research has been completed on feeding programs for pullets. For example, energy and protein (amino acids) requirements to optimize body development and composition at the point of lay (Leeson and Summers, 1981; Atkinson et al., 1991; Keshavarz and Jackson, 1992; Bahry et al., 2023). Moreover, the intimate linkage between the skeletal integrity of laying hens and medullary bone calcium reserves at the point of lay has elicited tremendous interest in mineral nutrition (Hurwitz and Bar, 1971; Keshavarz, 1987; Khanal et al., 2019, 2020a, 2021). It has also been postulated that pullets with well-developed digestive tract at the point of lay are likely to attain an upward of 40% increase in FI from the point of lay through to peak production (Leeson and Summers, 2005; Bain et al., 2016). In this context, incorporation of fibrous feedstuffs such as oat hulls to stimulate gastrointestinal tract development in pullets has also been investigated (Guzmán et al., 2015; Saldaña et al., 2015).

Poultry gastrointestinal tract particularly ceca harbors exceedingly complex microbiota that serves many functions including immunometabolism, resilience to enteric pathogens, and animal productivity (Yadav et al., 2018). Gastrointestinal microbes are amenable to dietary components with implications on various animal states (Kiarie et al., 2013). For example, broad-spectrum antibiotics such as bacitracin with zero egg withdrawal time are often added to diets for laying hens at subtherapeutic levels to manage stress related to heat, feed change, enteritis, etc. (Manner and Wang, 1991; AAFC, 1994; Huyghebaert and De Groote, 1997; Singer et al., 2023). However, there are concerted efforts to investigate alternatives to the use of subtherapeutic antibiotics. Probiotics are well documented in terms of modulating the gut microbiota, immune system, nutrients utilization, and performance in poultry (Neijat et al., 2019a, b; Mak et al., 2022). Feeding pullets probiotic during rearing reduced stress associated with phase feeding (stage-to-stage diet transition) and the benefit could be seen in higher BW and uniformity in probiotic-fed pullets (Neijat et al., 2019c). Studies in laying hens indicated supplemental medium chain fatty acids (**MCFA**) such as myristic acid (**MA**) increased egg production and quality indices linked to modulation of gastrointestinal microbiota and fat metabolism (Zhao et al., 2019; Liu et al., 2020). Through lowering gastrointestinal pH, organic acids have been associated with facilitating calcium solubility and absorption for increased eggshell quality (Świątkiewicz et al., 2010; Cao et al., 2022). Organic acids have also been demonstrated to exhibit broad antimicrobial activity against many gastrointestinal pathogens (Knarreborg et al., 2002; Friedman et al., 2003).

There is a dearth of data on the effects of supplementing feed additives to pullets at the onset of lay and the subsequent impact on egg production, nutrient utilization, metabolism, and gastrointestinal ecology. However, ongoing changes in hen housing and restriction on the use of preventative antibiotics could increase the risk of proliferation of pathogenic and zoonotic bacteria in the gut (Kaufmann-Bart and Hoop, 2009; Ngunjiri et al., 2019; Adhikari et al., 2020; Bean-Hodgins and Kiarie, 2021; Joat et al., 2023). Indeed, a recent detailed analyses of lifetime (hatch to end of lay) microbial shifts in excreta samples of four commercial layer flocks from different housings demonstrated significant shedding of *Escherichia*, *Campylobacter*, and *Clostridium* species at the onset of lay (Joat et al., 2023). It is therefore imperative to evaluate the impact of dietary strategies in modulating gastrointestinal ecology at the onset of lay. The objective of the present study was to investigate FI, egg production, egg quality, ceca digesta microbial activity, and apparent retention (**AR**) of components in Lohmann LSL Lite pullets fed selected feed additives (MA, BA, and *Aspergillus niger* probiotic) from the point of lay to 31 weeks of age (woa). An additional diet treated with bacitracin was used for comparative evaluation.

## MATERIALS AND METHODS

The University of Guelph Animal Care approved animal care and user protocol (#4518), and Use Committee and birds were cared for following the Canadian Council on Animal Care guidelines (CCAC, 2009).

### Birds and Housing

The study utilized 1,200 Lohmann LSL Lite layer birds that were 18 **woa** at the commencement of the study. The birds had been sourced from a commercial farm (Archer’s Hatchery, Brighton, Ontario, Canada) as day-old chicks and reared in accordance with Lohmann management guide. At 18 woa, experimental birds were randomly selected and reallocated into the study rooms. The birds were placed in 40 enriched layer cages (30 birds/cage) based on 18 woa BW. The cages were housed in three rooms at the Arkell Poultry Research Station (Arkell, ON, Canada) as previously described by Khanal et al. (2020b). Briefly, the dimensions of each cage were 365 cm × 65 cm × 55 cm (L × W × H) and was equipped with a feeder (365 cm × 8.5 cm × 15 cm, L × W × H) at the front. Two parallel perches, 20 cm apart, of 10 cm in circumference and 240 cm in length were placed in the mid region of cage and installed at 8 cm above the floor. Each cage was equipped with 12 nipple drinkers placed equidistantly along the midline of the cage and a nest (60 cm × 30 cm × 55 cm, L × W × H). The average space and total utilizable space per pullet in the furnished cage were 1,186 cm^2^ and 23,725 cm^2^, respectively. The room temperature was maintained at between ~18 and 20 °C throughout the entire trial. The birds received 14 h of incandescent light per day (0300 to 1700 h), with no humans in the room within the first 4 h of light to allow for an undisturbed lay period for the hens. Light intensity was kept at 15 lux.

### Feed Additives and Experimental Diets

A basal diet (Table 1) was formulated to meet or exceed the nutrient specifications of LSL lite hens (Lohmann, 2016). The test diets were made as follows: 1) bacitracin methylene disalicylate (**BMD)**, basal plus 1 kg of BMD 110 G per ton to provide 110 mg BMD per kilogram of feed (Zoetis Canada Inc., Kirkland, QC, Canada); 2) MA provided by Gutmyria (≥30% MA) at 300 g per ton of feed; 3) BA provided by Benzocal-50 (≥50% BA) at 330 g per ton of feed; and 4) PRO (5 × 10^8^ spores/g) provided by Probioist at 200 g per ton. Bacitracin is approved for use in laying hens in Canada with veterinary prescription and with no egg withdrawal time so long as fed at a maximum of 110 g/kg (AAFC, 1994). The MA, BA, and PRO were procured from Guangzhou Insighter Biotechnology Co., Ltd, Guangzhou, China. The inclusion in the current study was based on the supplier recommendations. The diets were manufactured at the University of Guelph, Arkell Research Station. A total of three batches; 1 ton/batch of each diet were manufactured for the entire trial. The manufacturing sequence was as follows: Basal, MA, BA, PRO, and BMD with flushing with 500 kg of wheat between diets. The third batch of the diets had 0.25% TiO_2_ indigestible marker to facilitate the determination of AR of components. All diets were steam-pelleted at a temperate range of 65 to 70 °C; in a die with 410 mm in diameter, an effective press width of 114 mm, and area of 0.18 m^2^.

**Table 1.** Composition of the basal diet, as fed basis^1^

Ingredient	Amount
Corn	55.6
Soybean meal	15.0
Wheat	10.0
Pork meal	6.00
Limestone fine (≤0.595 mm)	4.91
Limestone coarse (≥2 mm)	3.68
Soy oil	2.27
Monocalcium phosphate	1.18
Vitamin and trace minerals premix^2^	0.50
Titanium dioxide	0.25
Salt	0.25
dl-Methionine	0.20
Sodium bicarbonate	0.15
l-Lysine HCl	0.03
l-Threonine	0.02
Calculated provisions	100.0
AMEn, kcal/kg	2,750
CP, %	17.00
Crude fat, %	5.59
Linoleic acid, %	2.29
Calcium, %	3.80
Available phosphorous, %	0.45
Sodium, %	0.18
Chloride, %	0.20
SID Lys, %	0.69
SID Met, %	0.42
SID Met + Cys, %	0.62
SID Trp, %	0.37
SID Thr, %	0.48

^1^The feed additive diets were made by top dressing the basal diet as follows: BMD, basal plus 1.0 BMD 110 G per ton  to provide 110 mg bacitracin methylene disalicylate per kilogram of feed; MA, myristic acid provided by 300 g of Gutmyria per ton; BA, benzoic acid provided by 330 g of Benzocal-50 per ton; PRO, *A. niger* provide by 200 g of Probioist per ton.

^2^Provided per kg of premix: vitamin A (retinol), 2,400 KIU; vitamin D_3_ (cholecalciferol), 1,000 KIU; vitamin E, 16 KIU; vitamin K_3_ (menadione), 660 mg; vitamin B_1_ (thiamin), 800 mg; vitamin B_2_ (riboflavin), 1,720 mg; vitamin B_3_ (niacin), 13,000 mg; vitamin B_5_ (pantothenic acid), 4,000 mg; vitamin B_6_ (pyridoxine), 860 mg; vitamin B_9_ (folic acid), 440 mg; vitamin B_12_ (cyanocobalamin), 3.4 mg; biotin, 44 mg; choline, 120,000 mg; Fe, 12,000 mg; Cu, 2,000 mg; I, 200 mg, Se, 60 mg; Mn, 14,000 mg; Zn, 14,000 mg; Ca, 158 g.

### Experimental Procedures and Samples Collection

The five diets were allocated to cages using an incomplete randomized block design to give eight replicates per diet. The incomplete design was occasioned by the fact that out of the three rooms, two rooms had 16 cages each and the third room had 8 cages. As such although all the diets were represented in all rooms, the number of replicates per diet across rooms differed. Birds had *ad libitum* access to feed and water. The eggs were recorded daily at 1000 h for calculation of hen-day egg production (**HDEP**). The FI was recorded as the difference between the feed added in the trough throughout the week and the feed remaining in the feeder at the end of the week. Beginning at 23 woa, nine eggs per cage were randomly selected for egg quality analyses on a biweekly basis (i.e., at 23, 25, 27, 29, and 31 woa). These eggs were subsequently subjected to analyses of eggshell breaking strength (**ESBS**) and thickness (**EST**), haugh unit (**HU**), and yolk color (**YC**) as described by Mwaniki et al. (2018) using Egg Tester Ultimate (ORKA Food Technology LLC, UT, USA). Briefly, the unit electronically measures and calculates egg weight, YC, HU, ESBS, and EST per egg using ultrasound in 17 s. The YC was based on DSM Yolk Color Fan (formerly Roche Yolk Color Fan). Mortalities were monitored throughout the trial. All the hens in the cage at the beginning of 18 woa and at the end of 31 woa were weighed to determine the average hen BW per cage. Grab samples of fresh excreta were collected on cage basis every Monday from 23 through to 31 woa for the determination of excreta moisture content as a gross indicator of gut health (Kiarie et al., 2009). Excreta samples were collected Monday through Thursday at 31 woa for determination of AR of energy and nutrients (Neijat et al., 2019b). The samples were stored at 4 °C during collection, thoroughly mixed after the last day of collection and a subsample transferred to −20°C until required analyses. At the end of the trial, two birds per cage were individually weighed and bled via the wing vein. The blood was drawn in heparin-coated tubes, placed on ice, and transferred to the laboratory for extraction of plasma. The bird was subsequently euthanized through cervical dislocation, abdominal cavity opened, the twin ceca excised, and all digesta samples from the two birds were emptied into a common sterile tube, placed on ice, and immediately transported to the laboratory.

### Sample Processing and Laboratory Analyses

The fresh excreta samples were placed in an oven at 60 °C for 72 h to determine moisture content (Kiarie et al., 2009). The excreta samples for AR of components were placed in an oven at 60 °C and dried to a constant weight. The dried excreta and diet samples were finely ground separately using a coffee grinder (CBG5 Smart Grind, Applica Consumer Products Inc., Shelton, CT, USA) and thoroughly mixed for chemical analyses. All samples were analyzed for dry matter (**DM**), ash, crude protein (**CP**), gross energy (**GE**), and titanium. The DM was determined using method 930.15 (AOAC, 2005) and nitrogen was analyzed using a CNS-2000 Carbon, Nitrogen, and Sulfur Elemental Analyzer (LECO Corporation, St. Joseph, MI, USA) method 2001.11 (AOAC, 2005). The CP values were derived by multiplying the nitrogen value by 6.25. The GE was determined using a bomb calorimeter (IKA Calorimeter System C 5000; IKA Works, Wilmington, NC, USA). Ash content was determined in a muffle furnace at 600 °C for 12 h (AOAC, 2005). Titanium concentration was measured using the method of Myers et al. (2004). The diet samples were further analyzed for the concentration of starch, crude fat, calcium, phosphorous, and sodium in a commercial laboratory (SGS Canada Inc, Guelph, ON, Canada).

Blood samples were centrifuged at 2,500 × *g*, 4 °C for 15 min to separate the plasma. Plasma samples were submitted to Animal Health laboratory (Guelph, ON, Canada) for analyses of a select panel of avian biochemistry profiles with the methods described by Greenacre et al. (2008). The ceca digesta samples were vortexed, and a subsample was taken for microbial analyses using BioFreeze kits (Alimetric Diagnostics Ltd., Espoo, Finland). Briefly, the kit had a preweighed vial loaded with preservation buffer and sampling scoop. The homogenized digesta was transferred to the scoop and the excess digesta was wiped away with a clean paper towel to ensure the scoop was evenly filled (approximately 1 g of digesta sample). The filled scoop was then placed back in the vial, tightly capped, and shaken vigorously to completely suspend the digesta into the preservation buffer. The vial was then turned upside down to detach all the digesta from the scoop. The vial and its content were subsequently shipped in room temperature conditions to Alimetric Diagnostics Ltd. for microbial analyses as described by Kiarie et al. (2021). Briefly, upon arrival at the laboratory, the vials were placed in a centrifuge and the supernatant was subjected to differential centrifugation to separate bacterial cells. The bacteria cells were then disrupted to extract DNA for 16S rRNA gene-targeted DistaMap microbial analyses (Tajadini et al., 2014) using quantitative real-time polymerase chain reaction. The present analyses targeted abundance of total bacteria, *Lachnospiraceae*, *Ruminococcaceae*, Bacteroides, *Bifidobacterium*, *Clostridium sensu stricto*, *Lactobacillus*, and *E. coli* with primers previously reported by Kettunen et al. (2017). The concentration of targeted bacteria was reported as copies of 16S RNA per gram of sample. The rest of ceca samples were stored at −20°C until required for determination of the concentration of short-chain fatty acids (**SCFA**). The samples were thawed and processed and analyzed for SCFA (acetic, propionic, isobutyric, butyric, isovaleric, and valeric) according to Leung et al. (2018).

### Calculations and Statistical Analyses

The daily FI (g/b/d) was calculated by dividing the cage FI value by the number of birds per cage at the end of the week. The HDEP (%), egg mass (**EM**, g/bird/d), FI (g/bird/d), and feed conversion ratio (**FCR**, FI divided by EM) were calculated as described by Mwaniki et al. (2018). The microbial data were log-transformed to base 10. The concentration values for *Lactobacilli* and *E. coli* were used for calculating *Lactobacilli*: *E. coli* ratio (**LER**). The AR of dietary GE, CP, and ash were calculated according to Kiarie et al. (2014). The data for the egg production and FI for 18 to 20 woa were considered early lay phase. The HDEP, FI, EM, and FCR between 21 and 31 woa were considered mid to peak lay phase. The rest of the data were presented based on measurement timepoint. Data were analyzed using the Proc Mixed procedure in SAS Enterprise Ed (v 9.4). The cage was the experimental unit; diet, time, and associated interaction were fixed factors. The block (room) was a random factor. Time was used in the repeated statement, using the autoregressive first order (AR (1)) covariance structure. The significance was declared at *P* ≤ 0.05 and trends (*P* ≤ 0.10) were discussed. Tukey procedure was used for means separation.

## RESULTS

The analyzed chemical composition in the experimental diets is shown in Table 2. In general, the analyzed chemical composition corresponded to formulated targets. Moreover, the chemical composition of experimental diets was consistent among the five dietary treatments.

**Table 2.** Analyzed chemical composition of experimental diets^1^

Item	Basal	Basal plus additives			
		BMD	MA	BA	PRO
DM, %	89.9	89.9	89.3	89.9	89.7
GE, kcal/kg	3,744	3,755	3,755	3,721	3,716
Starch, %	41.6	41.7	42.7	43.4	43.6
CP, %	17.2	16.9	17.1	16.9	16.7
Crude fat, %	5.11	5.25	4.88	5.32	5.31
Ash, %	12.3	14.4	13.1	12.7	13.9
Calcium, %	3.72	3.84	3.73	3.80	3.78
Phosphorous, %	0.62	0.66	0.62	0.59	0.64
Potassium, %	0.56	0.60	0.54	0.55	0.55
Magnesium, %	0.16	0.18	0.15	0.13	0.16
Sodium, %	0.17	0.19	0.18	0.17	0.18

^1^BMD, bacitracin methylene disalicylate; MA, myristic acid; BA, benzoic acid; PRO, *Aspergillus niger* probiotic.

### Performance

The egg production and FI data for the early lay phase period (18 to 20 woa) are shown in Table 3. There was no (*P* > 0.05) diet and woa interaction on HDEP or FI. However, egg production increased from 0.63% in 18 woa to 65.2% in 20 woa. There was a diet (*P* = 0.0003) and week (*P* = 0.001) effect on FI. In this context, birds fed BMD ate a similar amount of feed to BA birds, but more than birds fed basal, MA, and PRO. The BA and MA birds ate the same amount. The FI increased from 61 g/bird at 18 woa to 76 g/bird at 20 woa. The data for HDEP, FI egg weight, EM, and FCR from 21 to 31 woa (mid to peak lay phase) are shown in Table 4. There was an interaction (*P* = 0.023) between diet and period on HDEP, in this context, diet differences were observed for 21 to 23 woa period with basal birds having lower HDEP than birds fed feed additives. Overall (21 to 31 woa), basal birds had lower (*P* = 0.001) HDEP than birds fed feed additives but there were no differences between feed additives on HDEP during this period (Table 4). The HDEP was 95.4%, 96.7%, 97.6%, 96.9%, and 96.7% for basal, BMD, MA, BA, and PRO, respectively between 21 and 31 woa. There was no interaction (*P* = 0.104) between diet and period on FI; the diet effect (*P* = 0.022) was such that basal birds ate less than birds fed additives (Table 4). There was an interaction (*P* < 0.0001) between diet and period on egg weight (EW). Specifically, basal birds had lighter eggs than BMD, BA, and PRO in 21 to 23 woa than feed additive birds in 24 to 25, 26 to 27 woa than PRO in 28 to 29 woa but heavier eggs than feed additives birds in 30 to 31 woa. Overall, 21 to 31 woa, eggs of basal birds were lighter (*P* < 0.0001) than eggs of birds fed additives. Eggs of birds fed BMD and PRO had similar (*P* > 0.05) weight, however, BMD eggs were heavier than for MA and BA birds. Interaction between diet and period on EM was such basal birds had lower EM than feed additives in 21 to 23, 24 to 25, 26 to 27 woa. However, basal birds EM was similar (*P* > 0.05) to that for feed additives birds in 28 to 29 and higher (*P* < 0.05) than for BA or PRO birds in 30 to 32 woa (Table 4). The EM of basal birds was lower than that of birds fed feed additives, however, there were no differences between feed additives on EM in overall (21 to 31 woa). In general, eggs became heavier as birds aged. There was an interaction (*P* = 0.026) between diets and period on FCR. There was no diets effect on FCR in 21 to 23, 26 to 27, and 30 to 31 woa. Birds fed BMD showed lower FCR than birds fed MA, BA, and PRO in 24 to 25 woa and basal birds had better FCR than BMD MA, and BA birds in 28 to 29 woa. Overall (21 to 31 woa), there was no (*P* = 0.700) diet effects on FCR. The FCR was 1.878, 1.865, 1.883, 1.911, and 1.882 for basal, BMD, MA, BA, and PRO diets, respectively.

**Table 3.** Effects of feed additives on egg production and FI in LSL Lite hens from 18 to 20 woa

Item		HDEP^2^, %	FI, g/bird/d
Basal^1^		28.8	58.5^c^
Basal + BMD		26.3	79.1^a^
Basal + MA		28.6	62.0^bc^
Basal + BA		29.2	69.5^ab^
Basal + PRO		27.2	56.7^c^
SEM		1.08	3.83
woa			
18		0.63^a^	60.9^b^
19		18.1^b^	58.7^b^
20		65.2^c^	75.9^a^
SEM		0.84	2.98
Diet	woa		
Basal^1^	18	1.0	56.4
Basal + BMD	18	0.7	75.8
Basal + MA	18	0.5	59.3
Basal + BA	18	0.6	56.0
Basal + PRO	18	0.4	56.8
Basal^1^	19	19.2	47.5
Basal + BMD	19	16.8	76.5
Basal + MA	19	17.7	51.7
Basal + BA	19	20.9	77.9
Basal + PRO	19	16.1	40.1
Basal^1^	20	66.1	71.5
Basal + BMD	20	61.3	85.1
Basal + MA	20	67.4	75.0
Basal + BA	20	66.1	74.5
Basal + PRO	20	65.1	73.3
SEM		1.87	6.66
Diet		0.285	<0.01
woa		<0.01	<0.01
Diet*woa		0.711	0.116

^1^BMD, bacitracin methylene disalicylate; MA, myristic acid; BA, benzoic acid; PRO, *Aspergillus niger* probiotic.

^2^HDEP, hen-day egg production.

Within a column and factor of analyses, LSmeans assigned different letters assigned differs, *P* < 0.05. Data are means of eight replications per diet.

**Table 4.** Effects of feed additives on egg production, FI, EM and FCR in LSL Lite hens from 21 to 31 woa

Item		HDEP^2^, %	FI, g/b/d	Egg weight, g/b/d	EM, g/b/d	FCR, g/g
Diet						
Basal^1^		95.4^b^	102.5^b^	57.3^c^	54.7^b^	1.878
Basal + BMD		96.7^a^	106.1^a^	58.8^a^	56.8^a^	1.865
Basal + MA		97.6^a^	106.9^a^	58.1^b^	56.8^a^	1.883
Basal + BA		96.9^a^	107.8^a^	58.2^b^	56.4^a^	1.911
Basal + PRO		96.7^a^	106.6^a^	58.5^ab^	56.6^a^	1.882
SEM		0.375	1.209	0.137	0.238	0.023
woa						
21 to 23		93.1^b^	87.9^c^	54.8^d^	51.0^d^	1.730^d^
24 to 25		97.3^a^	112.5^a^	57.4^c^	55.8^c^	2.016^a^
26 to 27		97.0^a^	107.8^b^	58.4^b^	56.6^b^	1.908^b^
28 to 29		97.9^a^	114.7^a^	60.3^a^	59.0^a^	1.946^b^
30 to 31		98.0^a^	106.9^b^	60.1^a^	58.8^a^	1.818^c^
SEM		0.374	1.205	0.136	0.237	0.023
Diet	woa					
Basal^1^	21 to 23	89.1^b^	85.4	53.8^m^	47.9^o^	1.791^hijk^
Basal + BMD	21 to 23	93.5^f^	88.9	55.6^jk^	52.0^n^	1.719^jk^
Basal + MA	21 to 23	94.5^ef^	89.3	54.2^lm^	51.2^n^	1.750^ijk^
Basal + BA	21 to 23	93.8^ef^	88.9	55.5^jk^	52.1^mn^	1.711^jk^
Basal + PRO	21 to 23	94.8^def^	87.3	54.9^kl^	52.1^mn^	1.681^k^
Basal^1^	24 to 25	96.9^abcd^	104.9	55.1^jk^	53.4^lm^	1.966^bcdefg^
Basal + BMD	24 to 25	97.1^abc^	107.9	58.3^h^	56.6^hijk^	1.901^defgh^
Basal + MA	24 to 25	98.2^abc^	115.5	57.3^i^	56.3^ijk^	2.051^ab^
Basal + BA	24 to 25	97.2^abc^	117.6	57.2^i^	55.6^k^	2.116^a^
Basal + PRO	24 to 25	97.0^abcd^	116.8	58.9^fgh^	57.1^ghij^	2.048^abc^
Basal^1^	26 to 27	95.9^bcde^	107.6	55.9^j^	53.6^l^	2.011^abcde^
Basal + BMD	26 to 27	97.1^abc^	109.1	59.5^def^	57.7^efghi^	1.891^defgh^
Basal + MA	26 to 27	98.0^abc^	107.3	58.7^fgh^	57.5^fghij^	1.867^efghi^
Basal + BA	26 to 27	98.2^abc^	108.9	59.4^def^	58.3^bcdefg^	1.868^efghi^
Basal + PRO	26 to 27	95.6^cdef^	106.2	58.6^gh^	56.0^jk^	1.902^cdefgh^
Basal^1^	28 to 29	97.3^abc^	105.8	60.1^cde^	58.5^bcdefg^	1.812^hijk^
Basal + BMD	28 to 29	98.0^abc^	117.7	60.2^bcd^	59.0^abcde^	1.995^abcdef^
Basal + MA	28 to 29	98.2^abc^	117.2	60.6^bc^	59.5^abc^	1.971^abcdefg^
Basal + BA	28 to 29	97.7^abc^	117.9	59.5^def^	58.1^cdefgh^	2.032^abcd^
Basal + PRO	28 to 29	98.2^abc^	114.9	61.0^ab^	59.9^ab^	1.919^bcdefgh^
Basal^1^	30 to 31	97.5^abc^	108.9	61.7^a^	60.2^a^	1.811^hijk^
Basal + BMD	30 to 31	97.7^abc^	107.0	60.2^bcd^	58.9^abcdef^	1.819^hij^
Basal + MA	30 to 31	99.1^abc^	105.1	59.8^cde^	59.3^abcd^	1.774^hijk^
Basal + BA	30 to 31	97.5^abc^	105.8	59.4^defg^	57.9^defgh^	1.828^ghij^
Basal + PRO	30 to 31	98.0^abc^	107.8	59.2^efgh^	58.0^cdefgh^	1.860^fghi^
SEM		0.836	2.694	0.305	0.531	0.051
Diet		<0.01	0.022	<0.01	<0.01	0.700
Period		<0.01	<0.01	<0.01	<0.01	<0.01
Diet*period		0.023	0.104	<0.01	<0.01	0.026

^1^BMD, bacitracin methylene disalicylate; MA, myristic acid; BA, benzoic acid; PRO, *Aspergillus niger* probiotic.

^2^HDEP, hen-day egg production.

Within a column and factor of analyses (diet, woa, and interactions), LSmeans assigned different letters assigned differs, *P* < 0.05. Data are means of eight replications per diet.

### Egg Quality

There were interactions between diets and woa on ESBS (*P* = 0.001), EST (*P* < 0.0001), HU (*P* = 0.018), and YC (*P* = 0.001; Table 5). For ESBS, eggs of basal birds had similar ESBS to eggs of birds fed MA, BA, and PRO but higher than BMD birds at 27 woa. However, eggs of MA and BA birds had similar ESBS to eggs of basal and PRO birds but higher ESBS than eggs of BMD birds at 29 woa. At 31 woa, eggs of BMD birds had similar ESBS to eggs of PRO birds but higher than for eggs of basal, MA, and BA birds. At 23 woa, EST was higher for eggs of BMD birds than eggs of birds fed other diets. The eggs of MA birds had thicker (*P* < 0.05) shell than the eggs of BMD and BA birds in 25 woa. There were no diet effects on EST at 27, 29, and 31 woa. Overall (23 to 31 woa), there were no diet effects on ESBS (*P* = 0.828), and EST (*P* = 0.606) and these parameters declined as birds aged (*P* ≤ 0.01). Interaction (*P* = 0.018) between diet and woa on HU was such that eggs of BMD birds showed lower HU than eggs of birds fed other diets in 23 woa but higher value in 27 woa. However, there were no diet effects on HU in 25, 29, and 31 woa. There was a tendency (*P* = 0.068) on HU to decline with age. Interaction (*P* = 0.001) between diets and woa on YC was such that diets had no effects in 23 woa, however, eggs of BMD had lower YC than birds fed other diets in 25, 27, and 29 woa. However, eggs of PRO birds showed similar YC to eggs of MA and BA birds but higher than for basal and BMD. Overall (23 to 31 woa), eggs of PRO birds had higher YC than eggs of basal and BMD birds (*P* < 0.0001). YC increased (*P* < 0.0001) with bird age.

**Table 5.** Effects of feed additives on egg quality attributes in LSL Lite hens from 23 to 31 woa

Item		Eggshell		HU	YC
		Breaking strength, kg N	Thickness, mm		
Basal^1^		5.73	0.404	91.8	4.43^b^
Basal + BMD		5.65	0.406	91.1	4.04^c^
Basal + MA		5.68	0.402	90.8	4.53^ab^
Basal + BA		5.68	0.404	90.4	4.56^ab^
Basal + PRO		5.72	0.401	90.2	4.61^a^
SEM		0.057	0.003	1.836	0.064
woa					
23		5.93^a^	0.419^a^	90.1	4.15^c^
25		5.88^a^	0.421^a^	91.2	3.80^d^
27		5.83^a^	0.395^b^	95.3	4.35^b^
29		5.63^b^	0.395^b^	87.9	4.87^a^
31		5.59^b^	0.388^c^	89.9	5.00^a^
SEM		0.0571	0.004	1.836	0.064
Diet	woa				
Basal^1^	23	5.99^ab^	0.414^cde^	93.0^b^	4.19^fgh^
Basal + BMD	23	5.88^abc^	0.455^a^	75.2^c^	4.19^fgh^
Basal + MA	23	5.80^abcd^	0.406^defg^	94.2^ab^	4.22^fgh^
Basal + BA	23	5.93^ab^	0.410^cdef^	94.1^ab^	4.07^gh^
Basal + PRO	23	6.06^a^	0.408^defg^	93.9^b^	4.07^gh^
Basal^1^	25	5.92^ab^	0.421^bcd^	89.3^b^	3.96^h^
Basal + BMD	25	5.70^bcd^	0.408^def^	92.6^b^	3.25^i^
Basal + MA	25	5.95^ab^	0.413^cdef^	92.4^b^	3.94^h^
Basal + BA	25	5.89^abc^	0.435^b^	91.7^b^	3.92^h^
Basal + PRO	25	5.93^ab^	0.426^bc^	90.0^b^	3.92^h^
Basal^1^	27	5.94^ab^	0.398^efgh^	93.4^b^	4.40^efg^
Basal + BMD	27	5.70^bcd^	0.391^ghi^	105.4^a^	3.93^h^
Basal + MA	27	5.89^abc^	0.396^fgh^	92.6^b^	4.46^defg^
Basal + BA	27	5.88^abc^	0.400^efgh^	91.9^b^	4.51^def^
Basal + PRO	27	5.76^abcd^	0.391^ghi^	93.0^b^	4.44^efg^
Basal^1^	29	5.69^bcde^	0.399^efgh^	88.8^b^	4.85^bcd^
Basal + BMD	29	5.36^ef^	0.386^hi^	85.5^bc^	4.00^h^
Basal + MA	29	5.74^abcd^	0.400^efgh^	88.5^b^	5.07^abc^
Basal + BA	29	5.83^abcd^	0.398^fgh^	88.2^b^	5.14^ab^
Basal + PRO	29	5.54^de^	0.391^ghi^	88.4^b^	5.30^a^
Basal^1^	31	5.58^cde^	0.389^hi^	94.4^ab^	4.72^cde^
Basal + BMD	31	5.99^ab^	0.391^ghi^	96.7^ab^	4.85^bcd^
Basal + MA	31	5.57^cde^	0.393^ghi^	86.2^bc^	4.96^abc^
Basal + BA	31	5.09^f^	0.379i	86.1^bc^	5.15^ab^
Basal + PRO	31	5.72^bcd^	0.387i	85.8^bc^	5.30^a^
SEM		0.128	0.008	4.105	0.143
Diet		0.828	0.606	0.977	<0.01
woa		<0.01	<0.01	0.068	<0.01
Diet*woa		<0.01	<0.01	0.018	<0.01

^1^BMD, bacitracin methylene disalicylate; MA, myristic acid; BA, benzoic acid; PRO, *Aspergillus niger* probiotic.

Within a column and factor of analyses, LSmeans assigned different letters assigned differs, *P* < 0.05. Data are means of eight replications per diet.

### Ceca Digesta Microbial Activity, Excreta Moisture Content, and AR of Components

The effects of feed additives on the abundance of selected bacteria and concentration of SCFA are shown in Table 6. Birds fed BMD had a lower (*P* < 0.0001; 11.3 log_10_ 16SRNA gene copies/g) total bacteria abundance than birds fed feed additives (~13 log_10_ 16SRNA gene copies/g). Birds fed MA, BA, and PRO had a higher (*P* = 0.003) abundance of Bacteroides than basal and BMD birds. Hens fed PRO had a higher (*P* = 0.004) abundance of *Bifidobacterium* than birds fed other feed additives, but none differed with the control birds. The abundance of *Lactobacilli* was higher (*P* = 0.022) in hens fed PRO than in birds fed basal, BMD, or BA hens. The basal hens had a higher (*P* = 0.009) abundance of *E. coli* than birds fed BMD, BA, and PRO-treated feed. Consequently, BMD, BA, and PRO birds had a higher (*P* = 0.011) LER than hens fed the basal diet. Specifically, relative to basal-fed hens, the LER of the BMD, MA, BA, and PRO hens was 37%, 21%, 26%, and 45%, respectively. There were no diet effects (*P* > 0.05) on the concentration of ceca digesta SCFA (Table 6).

**Table 6.** Effects of feed additives on abundance of select bacteria and concentration of SCFA in the ceca digesta and AR (%) of components in 31-wk-old LSL Lite hens

Item	Basal^1^	Basal plus feed additives				SEM	*P*-Value
		BMD	MA	BA	+PRO		
Bacteria, log_10_ 16S rRNA gene copies/g							
Total bacteria	12.9^a^	11.3^b^	12.9^a^	12.8^a^	12.9^a^	0.09	<0.01
*Lachnospiraceae*	12.2	12.1	12.1	12.0	12.1	0.09	0.921
*Ruminococcaceae*	11.7	11.9	11.6	11.9	11.9	0.13	0.481
*Bacteroides*	11.0^b^	11.0^b^	11.4^a^	11.3^a^	11.3^a^	0.09	0.003
*Bifidobacterium*	10.6^ab^	10.4b	10.4^b^	10.3b	10.9^a^	0.12	0.004
*Clostridium sensu strictu*	7.77	7.17	7.30	7.07	6.98	0.28	0.320
*Lactobacilli*	11.5^b^	11.7^b^	12.0^ab^	11.6^b^	12.3^a^	0.18	0.022
*E. coli*	7.72^a^	5.87^b^	6.92^ab^	5.93^b^	5.80^b^	0.42	0.009
*Lactobacilli*: *E. coli* ratio (LER)	1.50^b^	2.06^a^	1.81^ab^	2.04^a^	2.18^a^	0.14	0.011
SCFA, µmol/g							
Lactic acid	13.0	15.1	22.6	18.9	18.5	4.70	0.645
Acetic acid	162.7	158.5	158.8	148.2	176.1	6.63	0.072
Propionic acid	42.6	43.5	44.0	39.4	43.7	2.82	0.769
Butyric acid	44.2	43.4	48.2	42.2	47.2	2.44	0.369
Isobutyric acid	17.0	15.8	16.1	16.3	12.8	1.14	0.096
Isovaleric acid	4.65	5.11	5.39	4.43	4.67	1.01	0.962
Valeric	4.34	5.28	4.57	4.29	4.52	0.40	0.456
Total SCFA^2^	288.6	286.6	299.6	273.8	307.5	11.31	0.276
Excreta moisture content^3^, %	77.3	76.2	76.4	75.4	74.9	0.638	0.070
AR, %							
GE	85.2^b^	85.5^b^	84.6^b^	85.4^b^	87.4^a^	0.433	0.002
CP	76.3^abc^	73.4^c^	71.9b^c^	77.6^ab^	78.6^a^	1.690	0.043
Ash	41.0^c^	50.7^b^	50.4^b^	48.4^b^	57.4^a^	1.597	<0.01

^1^BMD, bacitracin methylene disalicylate; MA, myristic acid; BA, benzoic acid; PRO, *Aspergillus niger* probiotic.

^2^Summation of lactic, acetic, propionic, isobutyric, butyric, isovaleric, and valeric acids.

^3^Excreta moisture was evaluated weekly between 23 and 31 woa; the LSmeans represent the overall (main effect of diet).

Within a row LSmeans assigned different letters assigned differs, *P* < 0.05. Data are means of eight replications per diet.

There was no interaction (*P* = 0.613) between the sampling week and diet on excreta moisture content, however, there was the main effect of sampling week (*P* < 0.001) on excreta moisture (data not shown). The excreta moisture ranged from 78.7% at 23 woa to 72.8% at 24 woa with excreta samples at 23, 26, and 30 woa showing higher moisture than samples collected at 24 and 29 woa. There was a tendency (*P* = 0.070) for the main effect of diet on excreta moisture with excreta samples; relative to birds fed basal diet, PRO birds had 3.2% less excreta moisture with birds fed other diets being intermediate (Table 6). Birds receiving PRO diet had higher (*P* < 0.05) AR of GE and ash than birds fed basal and other feed additives (Table 6). Birds fed BMD, MA, and BA had similar AR of ash but higher than for birds fed basal diet. The basal and PRO birds had similar AR of CP; however, the value for PRO birds was higher (*P* = 0.043) than for birds fed BMD, and MA (Table 6).

### Body Weight and Plasma Biochemistry

Overall, the final BW was 1,706, 1,648, 1,705 1,628, and 1,693 g/bird for basal, BMD, MA, BA, and PRO, respectively (Table 7). Plasma biochemistry profile is shown in Table 7. Birds fed basal diet had higher concentration of plasma protein (*P* = 0.01), amylase (*P* = 0.008), and lipase (*P* < 0.001) than birds fed diets with feed additives. The concentration of plasma globulin was lower (*P* = 0.047) for the birds fed BMD, MA, and PRO diets than birds fed basal diet. However, globulin concentration in birds fed BA was not different (*P* > 0.05) from that of birds fed other diets. The diets had no (*P* > 0.10) effects on plasma concentration of uric acid, glucose, cholesterol, and minerals.

**Table 7.** Effects of feed additives on BW and plasma biochemistry profile in 31-wk-old LSL Lite hens

Item	Basal^1^	Basal plus feed additives				SEM	*P*-value
		BMD	MA	BA	PRO		
BW, g/b							
Initial	1,203	1,188	1,220	1,197	1,197	12.6	0.176
Final	1,708	1,648	1,705	1,628	1,693	26.44	0.131
Total protein, g/L	53.3^a^	44.2^b^	44.1^b^	47.2^b^	45.3^b^	2.14	0.017
Albumin (A), g/L	19.9	17.3	17.1	18.6	18.2	0.81	0.133
Globulin (G), g/L	33.4^a^	26.6^b^	26.9^b^	28.6^ab^	27.1^b^	1.78	0.047
A:G ratio	0.650	0.654	0.638	0.647	0.671	0.03	0.948
Uric acid, μmol/L	213	243	243	209	239	17.7	0.460
Glucose, mmol/L	12.9	13.9	13.6	14.4	14.2	0.51	0.240
Cholesterol, mmol/L	3.60	3.06	2.74	3.52	2.94	0.34	0.318
Bile acid, μmol/L	43.4	32.3	36.0	38.7	38.3	4.37	0.478
Gamma-glutamyl transferase, U/L	42.1	77.7	43.6	79.1	82.1	19.7	0.391
Aspartate aminotransferase, U/L	148	162	136	151	149	10.4	0.569
Creatine kinase, U/L	991	1,083	865	875	954	90.8	0.430
Amylase, U/L	296^a^	244^b^	255^b^	246^b^	249^b^	11.4	0.008
Lipase, U/L	10.3^a^	6.69^b^	6.36^b^	6.69^b^	6.44^b^	0.63	<0.01
Lactate dehydrogenase, U/L	230	237	208	208	227	14.6	0.512
Calcium, mmol/L	6.82	6.40	5.98	6.84	6.52	0.37	0.499
Phosphorous, mmol/L	1.52	1.57	1.46	1.72	1.63	0.09	0.323
Sodium, mmol/L	149	149	148	139	149	4.34	0.353
Potassium, mmol/L	5.93	5.49	5.64	5.13	5.74	0.20	0.161
Chloride, mmol/L	112	114	111	104	113	3.27	0.197

^1^BMD, bacitracin methylene disalicylate; MA, myristic acid; BA, benzoic acid; PRO, *A. niger* probiotic.

Within a row LSmeans assigned different letters assigned differs, *P* < 0.05. Data are means of eight replications per diet.

## DISCUSSION

Between the onset of lay and peak production, the hen consumption capacity is limited for meeting the needs for egg production, growth, and maintenance. The feed additives tested in the present study increased FI relative to the control suggesting potential of modulating digestive capacity in the early phases of egg production. The increased FI was accompanied with increased HDEP and EM. Albeit with some notable differences perhaps related to differing mode of actions, there were implications of tested feed additives on the gastrointestinal ecology and host metabolism. As expected, FI, HDEP, FCR, EW, and EM increased with hen age (Hanlon et al., 2022). Similarly, eggshell quality indices (EST and ESBS) were expected to decline as the laying cycle progressed (Roland, 1979; Nys, 1986; Hanlon et al., 2022).

The BMD is a broad-spectrum antibiotic obtained by culturing *Bacillus Licheniformis* and is commonly used in laying hens with zero egg withdrawal time (AAFC, 1994; Singer et al., 2023). In agreement with the present study, feeding 28-wk-old broiler breeder pullets BMD for 32 wk increased HDEP, however, the EW was reduced and there were no effects on BW and mortality (Damron and Wilson, 1985). Laying quails fed BMD between 37 and 42 woa had higher egg production and better FCR; however, BMD had no effects on EW and FI (Manafi et al., 2016). In terms of egg quality, the same study reported BMD reduced albumen height and HU but increased eggshell weight and breaking strength. Studies have demonstrated that feeding MCFA increases egg production. For example, 40-wk-old Hy-Line Brown laying hens fed 300 mg of mixture of MCFA/kg of feed through to 64 woa produced more and heavier eggs than the control hens but had no effects on FI (Liu et al., 2020). MCFA are rapidly absorbed in the gastrointestinal tract and transported to the liver providing energy to support egg production (Wang et al., 2015). Feeding 100 to 200 g of BA/ton of feed to 45-wk-old Lohman pink-shell laying hens for 16 wk had no effects on HDEP, FI, and FCR (Gong et al., 2021). Utility of probiotics in poultry production is a well-established practice, however, much focus has been on probiotics of bacteria origin. *Aspergillus* is one of the fungi species that has been characterized in the gastrointestinal tract of poultry (Hume et al., 2012). Feeding 220 g/ton of PRO used in the present study to 45-wk-old Hy-Line W-36 white laying hens for 10 wk increased HDEP relative to BMD but had similar egg production to the control (Sharma et al., 2022). Similarly, feeding *Aspergillus awamori* spores (25 × 10^4^/g of feed) to 28-wk-old Hy-Line Brown for 6 wk increased HDEP and EM relative to the control (Saleh et al., 2017). However, unlike in the present study where the tested feed additives increased FI, other studies had contrary observations. For example, hens fed fungal probiotic ate significantly less feed than control (Saleh et al., 2017; Sharma et al., 2022). This could be because these studies used older birds (>28 woa) at the start of experimentation.

The internal and eggshell quality attributes are largely influenced by ample dietary supply of macronutrients such as protein/amino acids and calcium. A previous study with Hy-Line Brown hens at 40 woa indicated supplementation of a mixture of MCFA increased eggshell strength and thickness linked to increased circulating plasma calcium and alkaline phosphatase (Liu et al., 2020). Bovan brown hens fed MCFA from 26 to 70 woa showed increased eggshell quality (Świątkiewicz et al., 2010). These observations suggested that MCFA may be involved in calcium metabolism leading to increased eggshell attributes. Supplementation of BA had no effects on eggshell quality but increased HU and albumen height in 45-wk-old Lohman pink-shell laying hens (Gong et al., 2021). *Aspergillus niger* probiotic had no effects on eggshell quality in 45-wk-old Hy-LineW-36 but increased HU (Sharma et al., 2022). However, *Aspergillus awamori* probiotic fed to Hy-Line Brown at 28 woa increased EST. Gastrointestinal tract modifiers such as organic acids and probiotics are linked with increased nutrient digestion (Kiarie et al., 2018). A possible reason for better retention of ash in birds fed BMD and feed additives was linked to microbial activity influence on mineral absorption, particularly calcium. However, this increased retention of ash was not reflected in eggshell quality in the present study. This may be partly because the hens in the present study were still producing high-quality eggshell. For example, in our previous long-term trial (first egg to 100 woa) research, we did not observe a decline in eggshell quality attributes until 60 woa (Hanlon et al., 2022). The observed effects of feed additives on egg yolk were surprising and difficult to explain. However, although not measured in the current study, previous research indicated antibiotics can enhance the absorption of carotenoids (Lind et al., 2021).

The stability of gastrointestinal microbiota is critical for reducing risks of enteric disease, improving nutrient utilization, and therefore increasing performance. The ceca is the largest reservoir for the metabolically active microbiota in poultry and is amenable to dietary inputs. Indeed, dietary factors facilitate the colonization and proliferation of the opportunistic pathogens such as *E. coli* and *Clostridium* and suppression of gut health-promoting bacteria such as *Bifidobacterium* and *Lactobacilli* (Kiarie et al., 2013; Ducatelle et al., 2018; Joat et al., 2023). It is thus no coincidence that BMD birds had lower abundance of total ceca bacteria. The present study extended numerous studies that showed MCFA (Liu et al., 2020, 2023), BA (Gong et al., 2021; Zhang et al., 2022), and *Aspergillus* spp. based probiotics (Saleh et al., 2017; Sharma et al., 2022) created a favorable gut ecology for the commensal bacteria to thrive at the expense of pathogenic bacteria. For example, feeding mixed MCFA to laying hens reduced the abundance of genus *Bacteroides* and *Lachnospiraceae* but increased the abundance of *Rikenellaceae* and *Collinsella* groups and reduced mortality (Liu et al., 2023). Sharma et al. (2022) observed reduction in the concentration of *Clostridium perfringens*, *Salmonella* spp., and *E. coli* in the ceca digesta of laying hens fed *Aspergillus niger* probiotic. Reduction of the abundance of *E. Coli* and increase of *Bifidobacterium* and LER suggested feed additives tested in the present study increased indices of gut health. Pathogenic bacteria such as *E. coli* reduce nutrients available to support animal performance through direct uptake of the nutrients and/or increasing mucosal inflammation and cell turnover triggering increased utilization of nutrients for maintenance (Dibner and Buttin, 2002). Saccharolytic fermentation in the ceca mainly results in acetate, propionate, butyrate, H_2,_ and CO_2_ (Macfarlane and Macfarlane, 2003; Tiwari et al., 2019), and proteolytic fermentation yields harmful metabolites such as 2-methylbutyrate, isobutyrate, isovalerate, and phenols, amines, and CO_2_ (Brestenský et al., 2017; Feng et al., 2018; Tiwari et al., 2019). Although we did not observe strong effects of diets on the concentration of ceca digesta SCFA, PRO birds had tendency for increased higher acetic acids and lower isobutyric acid suggesting overall improvement of gut health.

Besides limiting performance, a consequence of limited FI at the onset of lay is the negative nutrient balances that can affect liver and bone metabolism. Liver metabolism can be affected due to the mobilization of body fat, and bone metabolism can be affected when more calcium is needed for eggshell formation (Leeson and Summers, 2005). With respect to modern hyperprolific laying hens, fat metabolism is critical for hepatic fitness (Robinson and Kiarie, 2019). The present study investigated several plasma metabolites as indicator of the impact of tested feed additives on metabolism. Plasma biochemical parameters are influenced by multitude of factors including age, sex, nutritional and health status, breed, season, and stress (Thorn, 2000; Cooper et al., 2014; Abeni et al., 2018). A previous study in laying quails indicated that BMD reduced serum glucose, cholesterol, triglycerides, and alkaline phosphatase but increased gamma-glutamyl transferase (Manafi et al., 2016). We did not observe differences in the concentration of uric acid, glucose, minerals, and fat-related metabolites perhaps indicating balanced supply of energy, amino acids, and minerals for metabolism. Plasma proteins and globulin are mainly synthesized in the liver and have many functions including maintaining colloidal status of the blood, molecules transportation, and immune system among many others (Harr, 2002). However, higher concentration of plasma total protein might be an indicator of inflammation (Harr, 2002). An often-observed effect of feeding MCFA and probiotics in laying hens is on indices of lipid metabolism indicated by the concentration of blood cholesterol and lipase (Mohan et al., 1995; Saleh et al., 2017; Liu et al., 2020). Although we did not observe feed additives’ effects on cholesterol, plasma lipase reduced in response to feed additives. In other studies, laying hens fed mixture of MCFA exhibited elevated serum total cholesterol, triglycerides, and high-density lipoprotein cholesterol (Liu et al., 2020). Marked differences in fat metabolism of broiler chickens fed MCFA were linked to reduced serum cholesterol and increased lipase activity (Shokrollahi et al., 2014; Wang et al., 2015). Whereas other studies reported no effects of MCFA on blood lipid metabolites in broiler chickens (Fortuoso et al., 2019). Such inconsistencies in responses may be indicative of differences in dietary, animal, and other environmental factors but do point to the possibility of using MCFA to modulate fatty acids metabolism in poultry species and as such further research on the utility of MCFA in laying hen nutrition warrant further investigations.

From sustainability and profitability perspective, there is increasing interest in prolonging the laying cycle to more than 100 woa or 500 eggs per hen without molting (Bain et al., 2016). Suggesting that supporting hen capacity at the onset of lay is becoming even more important. At the onset of lay, the bird is not only adjusting to her new environment, but she must consume enough energy and nutrients for her BW development and to reach the high peak in egg production. It is imperative to increase their FI from the end of the growing period toward the peak of production in a short time. The present data indicated that supplementing diets of pullets with antibiotic growth promoter (BMD) or alternatives (MA, BA, and PRO) had a positive impact on FI and egg production through modulation of indices for gut health and metabolism. Some feed additives may not only impact microbiota ecology but could also enhance immunometabolism that could benefit pullets in tackling stressors associated with the onset of lay. These findings offered potential strategies for transitioning pullets at the onset of lay to promote FI and egg production. However, the mechanism and optimum inclusion levels of the feed additives require further exploration and capture the entire laying cycle.

## Abbreviations

AR: apparent retention
BA: benzoic acid
BMD: bacitracin methylene disalicylate
BW: body weight
EM: egg mass
FCR: feed conversion ratio
FI: feed intake
HDEP: hen-day egg production
LER: Lactobacilli to *E. coli* ratio
MA: myristic acid
PRO: *Aspergillus niger* probiotic
woa: weeks of age
